# Pentoxifylline ameliorates non-alcoholic fatty liver disease in hyperglycaemic and dyslipidaemic mice by upregulating fatty acid β-oxidation

**DOI:** 10.1038/srep33102

**Published:** 2016-09-09

**Authors:** Jia-Hung Ye, Jung Chao, Ming-Ling Chang, Wen-Huang Peng, Hao-Yuan Cheng, Jiunn-Wang Liao, Li-Heng Pao

**Affiliations:** 1 Research Center for Industry of Human Ecology, College of Human Ecology, Chang Gung University of Science and Technology, Taoyuan, Taiwan; 2 Institute of Pharmacology, College of Medicine, National Yang-Ming University, Taipei, Taiwan; 3 Liver Research Center, Division of Hepatology, Department of Gastroenterology and Hepatology, Chang Gung Memorial Hospital, Linko, Taiwan; 4 Department of Chinese Pharmaceutical Sciences and Chinese Medicine Resources, College of Pharmacy, China Medical University, Taichung, Taiwan; 5 Department of Nursing, Chung Jen College of Nursing, Health Sciences and Management, Chia-Yi, Taiwan; 6 Graduate Institute of Veterinary Pathology, National Chung Hsing University, Taichung, Taiwan; 7 Graduate Institute of Health-Industry Technology, College of Human Ecology, Chang Gung University of Science and Technology, Taoyuan, Taiwan; 8 Department of Nutrition and Health Sciences, Chang Gung University of Science and Technology, Kweishan, Taoyuan, Taiwan

## Abstract

Nonalcoholic fatty liver disease (NAFLD), which includes simple steatosis, steatohepatitis, fibrosis, and cirrhosis, is characterised by abnormal fat accumulation in the liver in the absence of excessive alcohol intake. In patients with type 2 diabetes (T2D), concurrent NAFLD might increase the risk of chronic kidney disease and the mortality rate. Although several studies have examined the effectiveness of pentoxifylline (PTX) in NAFLD treatment, no results are available to verify the effectiveness of PTX in treating T2D associated with NAFLD. In this study, we developed a combined high-fat diet-induced obesity and low-dose streptozocin-induced hyperglycaemia mouse model to mimic the concurrent NAFLD and T2D pathological condition. By combining physiological assessments, pathological examinations, metabolomics studies on blood, urine, and liver, and measurements of gene and protein expression, we elucidated the effectiveness and the underlying mechanism of action of PTX in the hyperglycaemic and dyslipidaemic mice. Our results revealed that PTX ameliorated NAFLD in the hyperglycaemic and dyslipidaemic mice by upregulating fatty acid β-oxidation. Furthermore, the glycolysis pathway and branched-chain amino acid-related pathways in these mice were restored by PTX.

Nonalcoholic fatty liver disease (NAFLD) includes simple steatosis, steatohepatitis, fibrosis, and cirrhosis, and in NAFLD, abnormal fat accumulation occurs in the liver in the absence of excessive alcohol intake^1^. Globally, NAFLD patient numbers have increased rapidly in the past 20 years, and the epidemic data have revealed NAFLD occurrence rates of 25–30% and 12.6–50% in USA and Asia (Japan, China, and South Korea), respectively^2^,^3^,^4^,^5^; furthermore, the occurrence rate of NAFLD in children was reported to be 3–10%^6^. These findings have made NAFLD a major public-health concern.

NAFLD in patients is correlated with an increase in metabolic diseases (obesity, insulin resistance (IR), and type 2 diabetes (T2D))^7^, and NAFLD is regarded as the manifestation of metabolic syndrome in the liver^8^. Accordingly, NAFLD has been reported in 75% and 50% of patients with T2D and dyslipidaemia, respectively^9^. Moreover, T2D progression is correlated with the severity of NAFLD and IR^10^, and patients with concurrent NAFLD and T2D carry an increased risk of developing chronic kidney disease (CKD, 2-fold increased)^11^ and present an elevated death rate (2.2-fold)^12^. There is no available pharmacological treatment for NAFLD^13^; current management of NAFLD involves lifestyle changes, such as weight reduction, prevention of weight gain, intake of healthy diet, and performing regular physical activity^14^. Thus, effective drugs or compounds for treating patients with both T2D and NAFLD are urgently required.

Pentoxifylline (PTX) is a methylxanthine compound and a non-selective phosphodiesterase inhibitor that produces vasodilatory effects^15^. PTX was initially used to treat intermittent claudication and then was broadly used to treat peripheral vascular disease, ischaemic injury, and liver-related injuries such as liver ischaemia and reperfusion^15^, alcoholic hepatitis^16^, and nonalcoholic steatohepatitis (NASH)^17^. The common pharmacological mechanism of PTX was regarded as the inhibition of tumour necrosis factor-α (TNF-α) synthesis^18^, and because (1) TNF-α plays a critical role in obesity and IR^19^ and (2) PTX can inhibit TNF-α synthesis, several small clinical trials have been performed to verify the effectiveness of PTX in NAFLD treatment^20^. Although these clinical results have shown that PTX markedly lowers the levels of glutamate oxaloacetate transaminase (GOT) and glutamate pyruvate transaminase (GPT) and may ameliorate liver histopathological changes in NASH patients, further large and well-designed studies are necessary to confirm the observed effects^20^.

Under normal conditions, fatty acid β-oxidation in mitochondria plays a key role in energy homoeostasis^21^, and mitochondrial dysfunction, which results in a reduction of fatty acid β-oxidation, is a critical feature of NAFLD^22^. IR induced by obesity and fatty liver could inhibit peroxisome proliferator-activated receptor-α (PPAR-α) and lead to the reduction in β-oxidation^23^. PPAR-α is a nuclear receptor and transcription factor that regulates several genes related to fatty acid β-oxidation, such as genes encoding carnitine palmitoyltransferase 1 (CPT1) and medium-chain acyl-CoA dehydrogenase (MCAD)^24^,^25^,^26^, and PPAR-α is considered to represent a potential therapeutic target for NAFLD^27^.

As mentioned earlier in this section, the risk of CKD development and mortality rate might be increased in patients with both NAFLD and T2D. Previously, PTX treatment was shown to produce beneficial effects against NAFLD, but no research results are currently available to verify the effectiveness of PTX in the treatment of concurrent NAFLD and T2D. In this study, we developed a combined high-fat diet (HFD)-induced obesity and low-dose streptozocin (STZ)-induced hyperglycaemia mouse model to mimic the NAFLD-plus-T2D pathological condition, and used this combined mouse model to estimate the effectiveness of PTX in the treatment of concurrent NAFLD and T2D. Moreover, we used a metabolomics strategy to delineate the possible pharmacological mechanism of PTX in this complex metabolic disorder.

## Results

### Establishment of the obesity, IR, and hyperglycaemia mouse model and evaluation of the benefits of PTX administration

**Figure 1:**
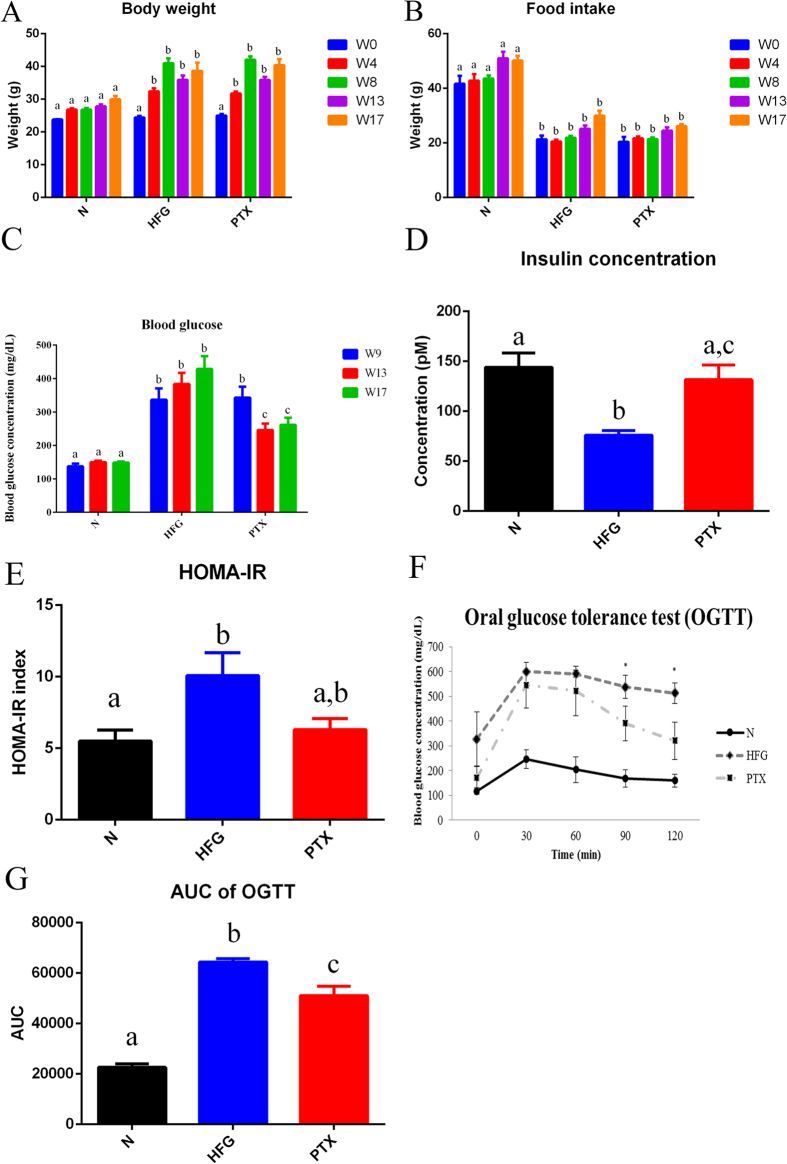
Physiological assessments of mice with combined NAFLD and T2D. The figure shows (**A**) body weight, (**B**) food intake, (**C**) blood-glucose levels, (**D**) insulin concentration, (**E**) homoeostasis model assessment-insulin resistance (HOMA-IR) index, **(F**) oral glucose-tolerance test (OGTT) results, and (**G**) area under the curve of OGTT of the normal group (**N**), HFD plus hyperglycaemia group (HFG), and PTX-treatment group (PTX). Values are presented as means ± SEM (N = 8); various letters indicate significant differences (*P* < 0.05).

To establish the combined NAFLD and T2D animal model, we modified the mouse model of HFD-induced obesity from our previous study^28^ and used low-dose STZ injection to induce hyperglycaemia in order to mimic the late stage of T2D^29^,^30^. The metabolic disorder of T2D was previously reported to be manifested following 3 injections of low-dose STZ in obese mice^30^. In our preliminary test, 2 injections of STZ (40 mg/kg) induced mild hyperglycaemia (>300 mg/dL) in obese mice without severe weight loss or death. HFD induced obesity in the HFD plus hyperglycaemia (HFG) group and the PTX-treatment (PTX; 100 mg/kg) group at Weeks 4 and 8 (W4 and W8), and after the 2 injections of low-dose STZ, slight body weight loss was observed (Fig. 1). However, at W13 and W17, body weight and food intake did not differ between the PTX group and the HFG group (Fig. 1A,B).

After the STZ injections, blood glucose was markedly elevated in the HFG and PTX groups at W9 (Fig. 1C), but PTX administration lowered the blood glucose level at W13 and W17 (Fig. 1C). The insulin concentration measured in the HFG group was almost half that in the control (normal) group, whereas insulin levels did not differ between the PTX group and the normal group (Fig. 1D). Furthermore, measurement of the HOMA-IR (homoeostasis model assessment-insulin resistance) index revealed marked IR in the HFG group, and PTX treatment moderately improved the IR (Fig. 1E). We also performed the oral glucose-tolerance test (OGTT) to evaluate the effect of PTX on glucose tolerance, and found higher blood-glucose control in the PTX group than in the HFG group at 90 and 120 min (Fig. 1F). The area under the curve of OGTT showed that PTX treatment significantly (*P* < 0.05) improved blood-glucose control (Fig. 1G).

In summary, the HFD/STZ mouse model developed here exhibited obesity, hyperglycaemia, IR, and poor glucose tolerance and thus mimicked the pathophysiological changes of T2D, and administration of PTX (100 mg/kg) alleviated hyperglycaemia and improved glucose tolerance and mitigated IR in the HFD/STZ mice.

### Histopathological examinations revealed that PTX treatment ameliorated the fatty liver in the combined NAFLD and T2D mouse model

**Figure 2:**
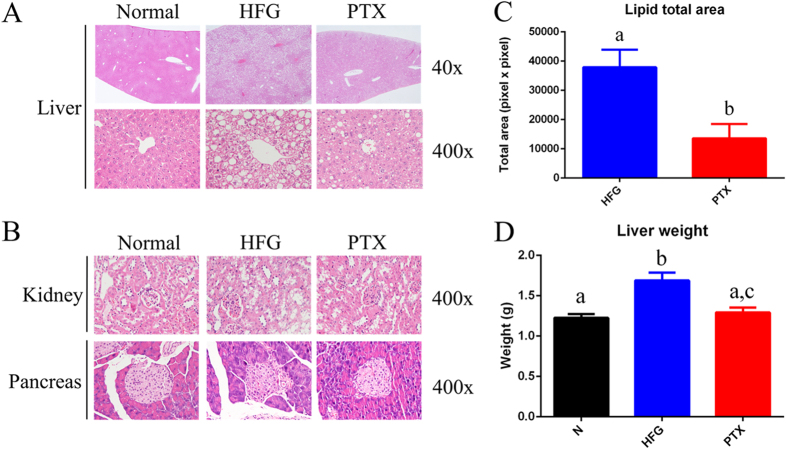
Pathological examinations of mice with combined NAFLD and T2D. Histopathological analysis of sections of (**A**) liver and (**B**) kidney and pancreas, showing the steatosis, lipid accumulation, and pancreatic atrophy in the HFG group, respectively; PTX administration ameliorated these pathological changes. (**C**) The lipid area of lipid vacuoles in the HFG and PTX groups, calculated using ImageJ, under 100× magnification (N = 6). (**D**) Liver weight measured for the 3 groups (N = 8). Values are presented as means ± SEM; various letters indicate significant differences (*P* < 0.05).

The HFG group exhibited considerable microvesicular and macrovesicular steatosis (Fig. 2A, Table 1). We observed more severe liver steatosis in the HFG group here in the combined NAFLD and T2D mouse model as compared with the liver steatosis induced by HFD alone^28^, which implies that hyperglycaemia and IR exacerbated liver steatosis. Moreover, in the HFG group, lipid accumulation was detected in the kidney, which reflected systemic metabolic disorder (Fig. 2B). Furthermore, the 2 injections of low-dose STZ and the systemic metabolic disorder might result in the atrophy and collapse of the islets of Langerhans (Fig. 2B, Table 1). We also calculated the liver lipid area based on microscopic analysis and thereby estimated the average lipid area in each histopathological section; the liver lipid area was significantly lower in the PTX group than in the HFG group, which indicates that PTX ameliorated lipid accumulation in the liver (Fig. 2C). Furthermore, liver weight was also restored to control levels after PTX treatment (Fig. 2D), and this might have resulted from the reduced lipid accumulation in the liver. Given the results of the histopathological examination and the liver lipid area and liver weight measurements, we conclude that PTX effectively ameliorated the fatty liver in our combined NAFLD and T2D mouse model.

Our results further showed that GOT, GPT, and triacylglycerol (TG) levels were lower in the PTX group than in the HFG group (Table 2). Elevated GOT and GPT in blood reflect liver damage, and increased blood levels of TG, cholesterol, and high-density lipoprotein (HDL) are key features of dyslipidaemia. To summarise, the model mice developed in this study were obese and hyperglycaemic and exhibited IR and diminished glucose tolerance, and the mice concomitantly presented fatty liver tissue, kidney lipid accumulation, pancreatic injury, liver injury, and dyslipidaemia; thus, these mice can serve as an adequate animal model for concurrent NAFLD and T2D, and in these mice, PTX administration effectively alleviated the metabolic disorders.

### Use of a metabolomics strategy to delineate the possible metabolic pathways restored in blood, urine, and liver following PTX treatment

**Figure 3:**
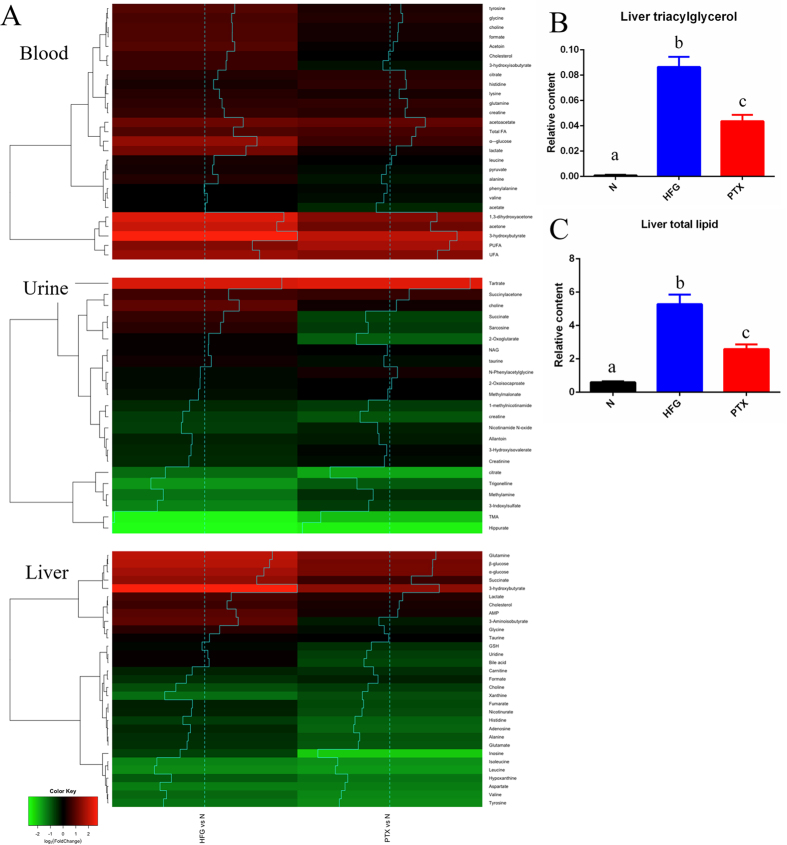
Fold-changes in metabolites in blood, urine, and liver. (**A**) Heatmaps of the metabolites in blood, urine, and liver. Hierarchical clustering is presented on the left side of the heatmaps, with traces added for easy reading (N = 8). (**B**) Liver triacylglycerol and (**C**) total liver lipid levels reflecting the severe fatty liver in the HFG group; PTX administration ameliorated the fatty liver. Values are presented as means ± SEM (N = 8); various letters indicate significant differences (*P* < 0.05).

In order to holistically investigate the possible metabolic pathways improved as a result of PTX treatment, we performed nuclear magnetic resonance (NMR) spectroscopy-based metabolomics studies on blood, urine, and liver. For comparisons, the HFG and PTX groups were separately normalised relative to the normal group. A total of 81 metabolites in the 3 distinct samples were identified, and the obtained heatmap revealed the fold-changes of the metabolites in the HFG and PTX groups (Fig. 3A).

First, relative to blood from the normal group, blood from the HFG group contained markedly higher levels of glycolysis-related metabolites (glucose and lactate), ketone bodies (KBs; 3-hydroxybutyrate), branched-chain amino acid (BCAA)-related metabolites (3-hydroxyisobutyrate), short-chain fatty acids (SCFAs; formate), glutamine, glycine, tyrosine, lysine, and cholesterol. Whereas glucose elevation indicates the hyperglycaemic state in the HFG group, KB elevation indicates the use of fatty acids as the energy source instead of glucose. Moreover, because 3-hydroxyisobutyrate can stimulate lipid uptake by skeletal muscle and lead to IR^31^, the increase in blood 3-hydroxyisobutyrate in the HFG group implies that skeletal muscle IR might occur in addition to hepatic IR; 3-hydroxyisobutyrate is an intermediate metabolite of the BCAA valine, and BCAAs were reported to spare glucose homoeostasis^32^ and were highly correlated with obesity-related IR^33^. Lastly, the gut microbiota can generate SCFAs (formate), and the SCFA level was previously correlated with obesity and NAFLD^34^. All of these abnormal elevations of metabolites in the blood of the mice in the HFG group implied that these mice not only developed IR in skeletal muscle and liver, but also harboured NAFLD-related gut microbiota, and thus the model mice presented the complete spectrum of the metabolic disorder. Notably, PTX administration led to a reduction in several of these abnormal metabolite elevations, such as those of glucose, lactate, 3-hydroxyisobutyrate, and formate, and thus ameliorated the aforementioned pathological changes.

Second, as compared to control, urine from the HFG-group mice showed a considerable reduction in the levels of 3 gut microbiota-related metabolites: 3-indoxylsulphate, hippurate, and trimethylamine (TMA). The decline in hippurate levels might be related to obesity, diabetes, and loss of renal function^35^. PTX administration increased 3-indoxylsulphate and also restored 3-hydroxyisoverate (an intermediate metabolite of the BCAA leucine) to control levels in urine.

Third, the HFG-group liver showed substantial increases (relative to normal liver) of the metabolites related to glycolysis and the tricarboxylic acid (TCA) cycle (glucose, succinate, and lactate), KBs (3-hydroxybutyrate), glutamine, and cholesterol, but a reduction in the levels of BCAAs (valine, leucine, and isoleucine), aspartate, tyrosine, and histidine. A concurrent comparison of the blood and liver metabolites revealed a positive correlation among the levels of glucose, lactate, 3-hydroxybutyrate, glutamine, and cholesterol, which implies that these metabolites in blood might be derived from the liver. By contrast, the BCAA level was lower in the liver than in blood, which indicates that skeletal muscle might play a critical role in BCAA release and degradation^36^. Here, PTX administration reduced the levels of succinate, glutamine, and glycine in the liver.

To verify the effectiveness of PTX in the treatment of fatty liver, we quantified liver TG and total lipids (Fig. 3B,C), and found that PTX administration markedly lowered liver TG and total lipid levels. Collectively, our results obtained using different methodologies (Figs 2A,C and 3B,C, Table 1) suggest that PTX ameliorated NAFLD in the combined NAFLD and T2D mouse model.

In summary, the metabolomics results implied that PTX treatment ameliorated NAFLD and T2D by restoring glycolysis and the TCA cycle pathway in the liver, BCAA metabolism in skeletal muscle, and the gut microbiota in the animal model presented here.

### PTX administration strongly induced the expression of genes related to fatty acid β-oxidation

**Figure 4:**
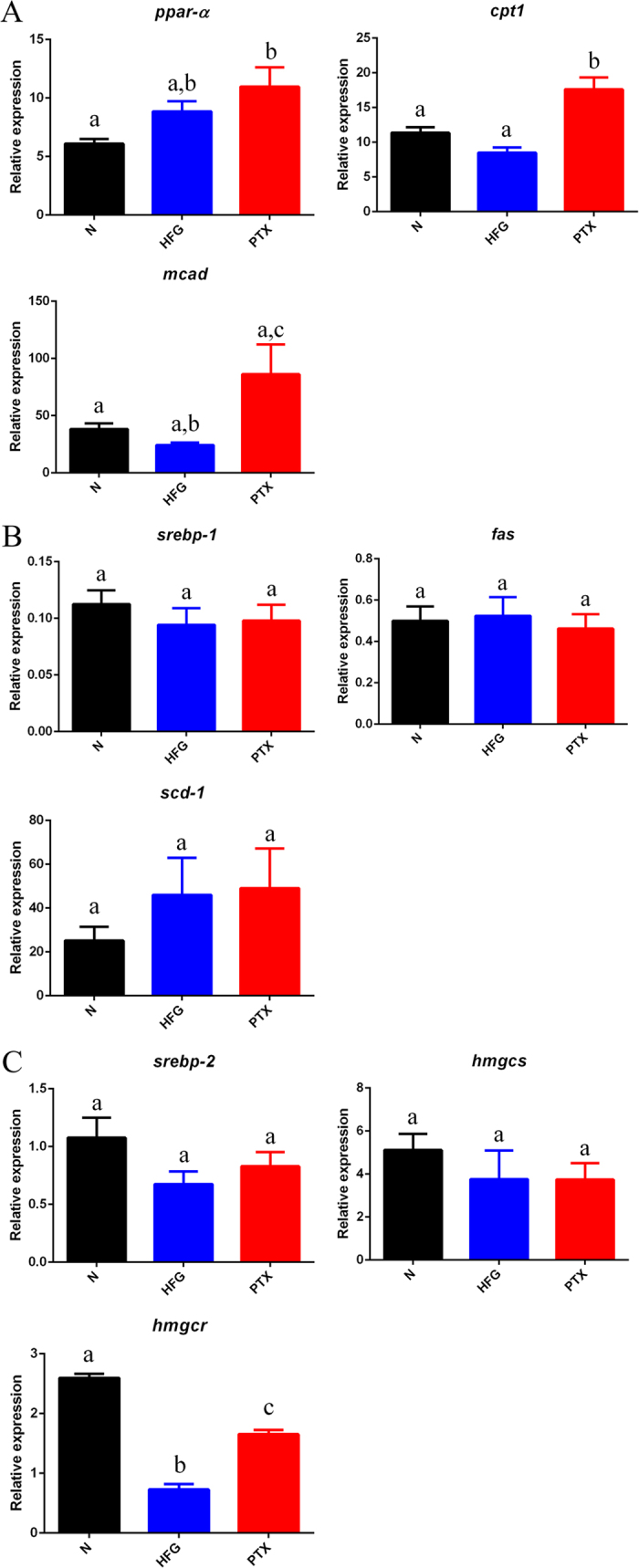
Expression of genes related to lipid metabolism. (**A**) Expression of genes related to fatty acid β-oxidation (*ppar-α, cpt1*, and *mcad*) in different groups; PTX administration upregulated *cpt1* and *mcad*. Expression of (**B**) lipogenesis-related genes (*serbp-1, fas*, and *scd-1*) and (**C**) cholesterol synthesis-related genes (*srebp-2, hmgcs*, and *hmgcr*) in different groups. Values are presented as means ± SEM (N = 6); various letters indicate significant differences (*P* < 0.05).

Next, to investigate the PTX pharmacological mechanism at a molecular level, we examined the expression of genes related to fatty acid β-oxidation, lipogenesis, and cholesterol synthesis. Because PTX administration ameliorated the fatty liver in the mice, we considered lipogenesis and fatty acid β-oxidation to be critical. Our results showed that *ppar-α* was expressed at moderately higher levels in the PTX group than in the HFG group, but that the PTX group showed substantial upregulation of the genes *cpt1* and *mcad* (Fig. 4A). CPT1 is crucial for fatty acid β-oxidation: the protein transports long-chain fatty acids into mitochondria for further catabolism. These results suggested that PTX might ameliorate fatty liver by upregulating genes related to fatty acid β-oxidation.

In contrast to the aforementioned results, no difference was observed in the expression of lipogenesis-related genes (*serbp-1, fas*, and *scd-1*) between the HFG and PTX groups (Fig. 4B); thus, PTX-dependent reduction in liver lipid content might result from an increase in fatty acid catabolism, rather than from the inhibition of lipid anabolism.

Intriguingly, as compared to the HFG group, the PTX group showed increased expression of the gene *hmgcr* (Fig. 4C); hmgcr is responsible for a key step in cholesterol synthesis and is the therapeutic target of statins. This increased expression of *hmgcr* coupled with the diminished level of cholesterol (Fig. 3A, liver) in the liver of mice in the PTX group is controversial and requires further investigation.

### PTX increased the expression of proteins related to fatty acid β-oxidation

**Figure 5:**
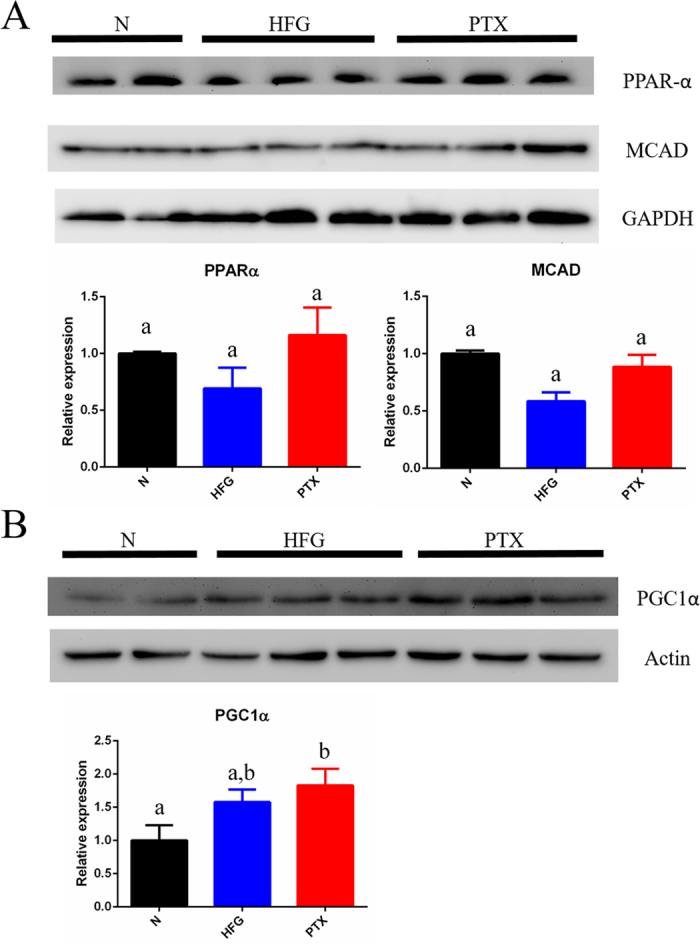
Expression of proteins related to fatty acid β-oxidation. Expression of (**A**) proteins related to fatty acid β-oxidation (PPAR-α and MCAD) and (**B**) PGC1α in different groups. The molecular weights of examined proteins were compared to those of pre-stained protein markers, and the membrane was carefully cropped. Values are presented as means ± SEM (N = 4–6); various letters indicate significant differences (*P* < 0.05).

Examination of the expression of proteins related to fatty acid β-oxidation revealed that PPAR-α and MCAD were moderately increased in the PTX group (Fig. 5A). Moreover, we measured the expression of Peroxisome proliferator-activated receptor gamma coactivator 1-alpha (PGC1α), because the protein not only plays a crucial role in regulating genes related to β-oxidation^37^, but is also responsible for mitochondrial biogenesis and oxidative metabolism^38^. Given that fatty acid β-oxidation occurs in mitochondria, we evaluated PGC1α protein expression to clarify the correlation between β-oxidation and mitochondrial biogenesis, and found that in the PTX group, PGC1α expression was higher than normal (Fig. 5B). The similar protein expression of PGC1α and gene expression of *ppar-α* suggested that PGC1α might function as a regulator upstream of or parallel to *ppar-α*. Furthermore, the increased PGC1α expression in the PTX group might reflect a more than normal number of mitochondria (the site of β-oxidation) and thus an increased requirement for elevated expression of genes related to fatty acid β-oxidation. In summary, PTX administration upregulated the expression of genes and proteins related to fatty acid β-oxidation in the combined NAFLD and T2D mice.

### Potential PTX pharmacological target pathways in the combined NAFLD and T2D mice

**Figure 6:**
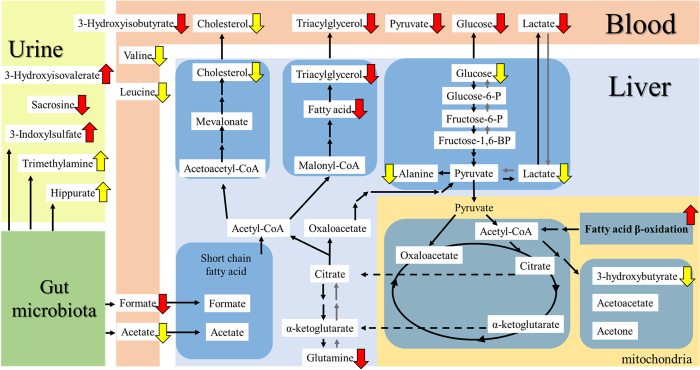
Summary scheme of PTX-targeted pathways in mice with combined NAFLD and T2D. The brief interactions between blood circulation and the liver are depicted. The gut microbiota might contribute to the systemic metabolic disorder. PTX administration could upregulate the expression of genes and proteins related to fatty acid β-oxidation and thereby ameliorate the fatty liver in the mice with combined NAFLD and T2D. Furthermore, the glycolysis pathway and BCAA-related pathways could be upregulated by PTX. The red and yellow arrows represent significant (*P* < 0.05) and moderate (*P* > 0.05) changes induced by PTX. The arrow direction indicates PTX-induced upregulation or downregulation.

Figure 6 presents a brief summary scheme of the PTX-targeted pathways that we identified in different samples. As shown by the results in Figs 1C and 3A and Table 2, PTX treatment markedly lowered the blood levels of the glycolysis-related metabolites glucose, lactate, and pyruvate, TG, the SCFA formate, and the BCAA intermediate metabolite 3-hydroxyisobutyrate, and moderately lowered the blood levels of cholesterol, BCAAs (valine and leucine), and the SCFA acetate. These results correspond with an alleviation of hyperglycaemia, glucose tolerance, and dyslipidaemia, and with a partial mitigation of skeletal muscle IR.

Following PTX treatment, the liver levels of fatty acids, TG, and glutamine were substantially decreased (Fig. 3A, liver), whereas the levels of glycolysis-related metabolites (glucose, lactate, and alanine), KBs (3-hydroxybutyrate), and cholesterol were moderately lowered. These results correspond with an improvement of fatty liver (microvesicular steatosis, lipid area, liver weight, and the content of liver TG and total lipids) and with a partial mitigation of hepatic IR. The underlying mechanism of action of PTX in the liver might involve an upregulation of genes and proteins related to fatty acid β-oxidation.

Interestingly, gut microbiota-related metabolites showed changes in the urine (3-indoxylsulphate, hippurate, and TMA) and blood (formate) from the PTX group, which implies that gut microbiota (the “forgotten organ”^39^,^40^) might play a key role in the systemic effectiveness of PTX. Although we performed systemic metabolomics studies on the blood, urine, and liver collected from the model mice, the lack of data from skeletal muscle, adipose tissue, and kidney might represent a drawback of this study. Our work also serves as a reminder that complex metabolic interactions occur in metabolic diseases.

Here, we have provided evidence indicating that PTX can ameliorate NAFLD in hyperglycaemic and dyslipidaemic mice. However, further clinical application of PTX in patients with concurrent NAFLD and T2D (who face an increased risk of death) remains to be investigated.

## Discussion

The present HFD/STZ mouse model exhibited obesity, hyperglycemia, IR, and poor glucose tolerance, and thus mimicked the pathophysiological changes of T2D. However, the HOMA-IR relies on fasting blood glucose levels for evaluation, and thus mostly describes hepatic IR and steady-state insulin secretion (a late marker of β-cell dysfunction)^41^. Therefore, HOMA-IR is not an adequate method to assess insulin sensitivity in the presence of β-cell damage, and the correlation between PTX and IR should be further carefully investigated.

Several studies have indicated that PTX can substantially lower GOT and GPT levels and ameliorates liver histopathological changes in NASH patients^20^,^42^. NASH is in the pathological category of NAFLD, but includes more criteria than simple steatosis does, and the degrees of steatosis, inflammation, and fibrosis provide crucial information regarding NASH. In the present study, the pathological examination for liver fibrosis was performed by Sirius Red staining (data not shown), and no fibrosis was observed in the livers of HFD/STZ mice. Except for a few Kupffer cells that were observed upon H&E staining, there was no indication of inflammation in the livers of HFD/STZ mice. Although severe steatosis was observed in this study, no inflammation or fibrosis was detected in the liver of mice in the HFG group. The major limitation of the present HFD/STZ mouse model is that it was unable to recapitulate the pathological condition of NASH, which could determine the prognosis of NAFLD. These results suggest that PTX could potentially be administered to patients with concurrent early liver steatosis and metabolic syndrome rather than NASH. Intriguingly, adverse effects of PTX treatment in NAFLD have also been reported: PTX was found to aggravate fatty liver in *ob/ob* mice^43^. The disparities in the animal model used (*ob/ob* vs HFD/STZ mice), the dosing vehicle (water vs HFD), and the treatment duration (3 vs 8 weeks) might be responsible for the previous results being completely different from our results. PTX treatment reduced steatosis and GOT levels in HFD-induced obese mice in a previous study^44^, which agrees with our findings; however, the PTX administration also lowered GOP levels and increased GPT levels in the obese mice as compared with the levels in normal mice, but the underlying reason was not provided. Nevertheless, our results revealed that PTX can ameliorate NAFLD in hyperglycaemic and dyslipidaemic mice. The role of PTX in blood-glucose control remains controversial, and several articles have reported that PTX cannot alleviate hyperglycaemia^43^,^44^, although PTX-dependent improvement was previously observed in hyperglycaemia in HFD-induced NAFLD Sprague-Dawley rats^45^, which agrees with our results. The complex and systemic nature of this metabolic disorder might be responsible for the incongruent results obtained with PTX. Notably, a previous meta-analysis revealed that caffeine (which possesses non-selective phosphodiesterase activity similar to that of PTX) has a protective effect against fibrosis in patients with NAFLD^46^. Given that both PTX and caffeine are methylxanthines and have beneficial effects against NAFLD, the methylxanthine class of drugs might have the potential to treat NAFLD.

In the HFG group in this study, lactate was substantially increased in blood and liver, and this could be because of increased synthesis of macromolecules (fatty acids and cholesterol). Our results showed that TG, lipid, and cholesterol levels in the HFG group were considerably higher than those in the normal group. The synthesis of these macromolecules requires a substantial amount of ATP and the cofactor NADPH. For example, the synthesis one molecule of palmitoyl-CoA requires 8 ATP and 14 NADPH molecules, and the synthesis of one cholesterol molecule requires 18 ATP and 26 NADPH molecules^47^. Under normal physiological conditions, the major source of ATP production is mitochondrial oxidative phosphorylation, which generates 36 molecules of ATP from one molecule of glucose^47^. However, mitochondrial dysfunction in NAFLD could result in impaired ATP production^22^. An imbalance develops when macromolecule synthesis requires ATP but mitochondrial dysfunction results in diminished ATP production, and for this problem, glycolysis might represent a solution: Glycolysis generates 2 ATP and 2 NADH molecules from one glucose molecule, and when glucose is abundant, the comparatively faster glycolysis process can generate more ATP than oxidative phosphorylation can^47^,^48^. In this study, glucose was found to be abnormally high in the liver of the HFG-group mice; thus, in these mice, we can expect lipids and cholesterol to be synthesised using the ATP generated from glycolysis rather than the impaired oxidative phosphorylation. The end product of glycolysis is pyruvate, which is converted into lactate by lactate dehydrogenase with the consumption of one NADH molecule^43^. Another role of lactate is to balance the NADH/NAD^+^ ratio in a cell: The greater the requirement of glycolysis-produced ATP for macromolecule synthesis, the higher the amount of NADH accumulated in the cell, and the elevated NADH/NAD^+^ ratio in the cell could drive the production of lactate^43^. Furthermore, the impaired oxidative phosphorylation resulting from mitochondrial dysfunction might contribute to NADH accumulation in the cell, which again would drive lactate production. As mentioned at the beginning of this paragraph, NADPH is required for macromolecule synthesis. In a cell, NADPH is produced by the pentose phosphate pathway (PPP) and from two conversions, isocitrate to α-ketoglutarate (αKG) and malate to pyruvate^43^. All three of these major sources of NADPH production are correlated with glucose levels, and thus the glucose present in an increased concentration in the liver of the HFG-group mice might produce the increased amounts of NADPH required for macromolecule synthesis. In summary, elevated levels of lactate in the liver might result from (1) the requirement of ATP for macromolecule synthesis, (2) the NADH/NAD^+^ ratio, and (3) the requirement of NADPH for macromolecule synthesis. The elevated lactate level in the circulation in the HFG group might imply that increased macromolecule synthesis was also required in skeletal muscle (3-hydroxyisobutyrate-stimulated lipid uptake)^31^ and kidney (lipid accumulation, Fig. 2B). However, the circulating lactate level was effectively lowered in the group treated with PTX, which is in accord with aforementioned points of view.

In the HFG group, the glutamine level in blood and liver was also elevated. Glutamine is a major carbon and nitrogen source and a contributor for anaplerosis in the cell^43^. Glutamine is converted into αKG by GOT, and αKG is not only a TCA cycle intermediate, but also a precursor of cytosolic acetyl-CoA (Fig. 6). Glutamine increase in the liver in the HFG group might be accompanied by a coordinated increase in cytosolic acetyl-CoA, and this could underlie the fatty liver observation. Future studies should investigate the correlation between increased circulating levels of glutamine and kidney lipid accumulation. Glutamine can be converted by glutaminase into glutamate, which exerts direct toxic effects on pancreatic β-cells^49^. Thus, increased levels of circulating glutamine might contribute to the atrophy of the islets of Langerhans in the HFG group (Fig. 2B). However, PTX administration was able to reduce liver glutamine, which is in line with the ability of PTX to ameliorate fatty liver and pancreatic atrophy in the hyperglycaemic and dyslipidaemic mice. In addition, the improvement of hyperglycaemia and metabolic disorder by PTX might result from alleviation of pancreatic atrophy and β-cell damage. The higher insulin concentration in the PTX group than in the HFG group might partially have contributed to the mitigation of metabolic disorder in the HFD/STZ mice.

Although PTX was also reported previously to enhance the expression of PPAR-α, the explanation provided was compensatory adaptation in response to abnormal fat deposition^43^. In another study, the genes related to fatty acid β-oxidation that were upregulated in HFD-induced obese mice were reported, and these genes were initially upregulated (4 weeks) and then downregulated (10 weeks)^50^. In this study, the HFD feeding lasted for 17 weeks, and in the PTX group, the expression of *cpt1* and *mcad* was substantially increased (Fig. 4A), which revealed that PTX can upregulate genes related to fatty acid β-oxidation. These results confirmed that no compensatory adaptation occurred in response to abnormal fat deposition or HFD-induced short-term upregulation of genes related to fatty acid β-oxidation. To delineate the molecular pharmacological mechanism of PTX against β-oxidation, further *in vitro* studies should be performed.

The HFG group showed a marked downregulation of *hmgcr*, which is responsible for the rate-limiting step in cholesterol synthesis. This observation agreed with the results of Chan *et al*.^50^, who also used HFD-induced obese mice and found that *hmgcr* was downregulated at Week 4; Chan *et al*. suggested that the liver was attempting to maintain cholesterol homoeostasis in response to HFD feeding, which resulted in the downregulation of *hmgcr.* At Week 10, *hmgcr* was upregulated and the circulating cholesterol level was increased. In this study, *hmgcr* was downregulated in the HFG group, which might have resulted from the maintenance of cholesterol homoeostasis in the liver (Fig. 4C). PTX administration markedly upregulated *hmgcr*, which might be correlated with the moderate reduction in circulating cholesterol. However, further investigation is required to elucidate the mechanism underlying the regulation of genes related to cholesterol synthesis.

We observed similar expression patterns for the protein PGC1α (Fig. 5B) and the gene *ppar-α* (Fig. 4A) in the 3 groups; thus, PGC1α might function upstream of or in parallel with *ppar-α*. However, the expression patterns of *ppar-α* (Fig. 4A) and the protein PPAR-α (Fig. 5A) were not identical in the HFG group as compared with control. Epigenetic regulation might occur during the translation of *ppar-α* mRNA, and, notably, microRNAs (miRNAs) play a key role in the inflammation and fibrosis in NAFLD, IR, and metabolic syndrome^51^,^52^. PPAR-α-related miRNAs, including miR-16, miR-33a/b, miR-200a/b, miR-181a, miR10b, and let-7b, were correlated with NAFLD^53^, and uncovering the functions of these miRNAs might provide new insights into the molecular mechanism of the PTX pharmacological effect in hyperglycaemic and dyslipidaemic mice.

The use of a metabolomics strategy can provide a holistic view of the metabolic status in a given scenario, and the usefulness of an integrated systems biology approach can be enhanced by combining transcriptomics, proteomics, metabolomics, and metagenomics. The combined NAFLD and T2D mouse model established here not only presents complex organ and/or tissue interactions: the model is also suitable according to several clinical criteria for metabolic diseases (obesity, IR, metabolic syndrome, NAFLD, and T2D). By combining physiological assessments, pathological examinations, metabolomics studies on blood, urine, and liver, and gene- and protein-expression analyses, the effectiveness and the underlying mechanism of action of PTX in hyperglycaemic and dyslipidaemic mice were uncovered in this study: PTX ameliorated NAFLD in the hyperglycaemic and dyslipidaemic mice by upregulating fatty acid β-oxidation, and, furthermore, PTX also restored glycolysis and BCAA-related pathways.

## Materials and Methods

### Animal model and treatments

In this study, 24 male 7-week-old C57BL/6J mice (BioLasco Taiwan Co., Ltd., Taipei, Taiwan) were used with approval from the Institutional Animal Care and Use Committee (IACUC) of Chang Gung University of Science and Technology (IACUC-2014-009). The housing, care, diet, and maintenance of the experimental animals were consistent with the recommendations of the National Research Council’s Guide for the Care and Use of Laboratory Animals, Animal Welfare Act, and Chang Gung University of Science and Technology Policy. All the studies was performed in accordance with IACUC guidance. For the control (normal) group, 8 mice were fed ad libitum on the normal diet (5001, LabDiet, MO, USA). For the experimental group, 16 mice were fed ad libitum on the HFD (58Y1, 60 kcal% fat, TestDiet, MO, USA) for 8 weeks to induce obesity, and then were intraperitoneally injected with STZ (40 mg/kg, 2 injections, Sigma) to induce hyperglycaemia (>300 mg/dL). The HFD/STZ animal model might be a suitable model for late-stage T2D^29^. One week later, 8 of the 16 mice in the experimental group of HFD plus hyperglycaemia were randomly selected and fed ad libitum on the HFD (HFG group), and to the remaining 8 mice, PTX (100 mg/kg, Sigma)^43^ was administered together with the HFD (PTX group); 8 weeks later, all mice were sacrificed and biospecimens (blood and tissue) were gathered and processed for further analysis.

### Body weight measurement and food intake

The body weight of all mice was measured initially (W0) and then once every 4 weeks (W4 and W8) after starting the HFD feeding. After STZ was injected to induce hyperglycaemia (W9), the body weight was again measured once every 4 weeks (W13 and W17). Food intake was recorded at the same time points.

### Blood-glucose measurement and OGTT

Blood glucose was measured from the tail blood at W9, W13, and W17 by using an Accu-Chek Glucometer (Roche). At W17, fasted mice were orally gavaged with glucose (1.5 g/kg), and blood glucose was measured at 0, 30, 60, 90, and 120 min after the glucose administration.

### Blood biochemistry

Blood was collected by performing cardiac puncture and then centrifuged at 4 °C, and the obtained plasma was used for measuring the levels of GOT, GPT, TG, total cholesterol, and HDL by using a Fuji DRI-CHEM 4000 (Fujifilm).

### Insulin measurement and HOMA-IR

Plasma insulin was measured using an ELISA kit (Millipore). HOMA-IR was calculated using this equation: HOMA-IR index = insulin (μU/mL) × glucose (mmol/L)/22.5^54^.

### Histopathological examination

The collected livers were weighed, and a part of the liver tissue was fixed in 10% formalin (Sigma). Next, conventional histological processing was performed, and then 4-μm-thick sections were cut for haematoxylin-eosin (H&E) staining. Lesion severity was graded according to the method described by Shackelford *et al*.^55^. The area of lipid vacuoles was calculated using ImageJ (National Institutes of Health, NIH), under 100× magnification^43^. Data were collected from 5 fields per mouse and 6 mice per group.

### Liver real-time polymerase chain reaction (RT-PCR)

Liver RNA was extracted using an RNeasy Plus Mini kit (Qiagen), and cDNA was prepared using a QuantiTech Reverse Transcription kit (Qiagen). The obtained cDNA was amplified by using TaqMan Probe (Applied Biosystems) in a StepOne Plus instrument (Applied Biosystems). The internal-control gene was *gapdh* (glyceraldehyde 3-phosphate dehydrogenase; assay ID Mm99999915_g1, Applied Biosystems), and the analysed genes were those encoding the following proteins: PPAR-α (assay ID Mm00440939_m1), CPT1 (assay ID Mm01231183_m1), MCAD (assay ID Mm01323360_g1), sterol regulatory element-binding transcription factor (SREBP)-1 (assay ID Mm00550338_m1), fatty acid synthase (FAS; assay ID Mm00662319_m1), stearoyl-CoA desaturase-1 (SCD-1; assay ID Mm00772290_m1), SREBP-2 (assay ID Mm01306292_m1), 3-hydroxy-3-methyl-glutaryl-CoA (HMG-CoA) reductase (assay ID Mm01282499_m1), and HMG-CoA synthase (assay ID Mm01304569_m1). Relative expression was quantified using StepOne 2.3 (Applied Biosystems).

### Immunoblotting

Protein expression was analysed by western blotting samples with antibodies against PPAR-α, MCAD, and PGC1α (GeneTex), following which semiquantitative image analysis was performed (MultiDoc-It Imaging System, UVP). (See also Supplementary Information)

### Metabolomics analysis of blood, urine, and liver

To delineate the PTX-targeted pathways, we performed NMR spectroscopy-based metabolomics analyses on various samples. Sample preparation, protocols, and data processing have been described previously^28^. We also generated heatmaps, which allow general two-dimensional visualisation with the information summary depicted using colours. Hierarchical clustering is one of the clustering algorithms used to compute the distance between each pair of samples in an analysis. In this study, dendrograms showing the hierarchical clustering of metabolite-expression data were generated by using heatmap.2 of the gplots package in R. As the default in heatmap.2 function, the Euclidean method is used to calculate the distance matrix for clustering. For the hierarchical clustering of data, the hclust function in R uses the Ward method.

### Statistical analysis

Data are presented as means ± SEM. The statistical significance of differences was examined by using one-way ANOVA with the Tukey multiple-comparisons test for post-hoc analysis; *P* < 0.05 was considered statistically significant. Nonparametric data were analysed using the Mann-Whitney test. Statistical analyses were performed using GraphPad Prism 6 (GraphPad) and SPSS (IBM).

## Additional Information

**How to cite this article**: Ye, J.-H. *et al*. Pentoxifylline ameliorates non-alcoholic fatty liver disease in hyperglycaemic and dyslipidaemic mice by upregulating fatty acid β-oxidation. *Sci. Rep.* **6**, 33102; doi: 10.1038/srep33102 (2016).

## Supplementary Material

Supplementary Information

## Figures and Tables

**Table 1:** Pathological examination of liver and pancreas in the 3 study groups.

	Liver		Pancreas
	Microvesicular steatosis	Macrovesicular steatosis	Atrophy
N	0.00	0.00	0.00
HFG	4.88 ± 0.35^a^	4.38 ± 0.74^a^	3.38 ± 1.30^a^
PTX	3.13 ± 0.35^b^	3.75 ± 0.89^a^	0.88 ± 1.64^b^

Abbreviations: N, normal group; HFG, high-fat diet plus hyperglycaemia group; PTX, high-fat diet/hyperglycaemia plus pentoxifylline treatment group. Lesion degree was graded from 1 to 5: 1 = minimal (<1%); 2 = slight (1–25%); 3 = moderate (26–50%); 4 = moderate/severe (51–75%); 5 = severe/high (76–100%)^55^. Values are presented as means ± SD (N = 8); superscripted letters indicate significant differences (Mann-Whitney test, *P* < 0.05).

**Table 2:** Blood biochemistry parameters in the 3 groups.

	GOT (U/L)	GPT (U/L)	TG (mg/dL)	TCHO (mg/dL)	HDL (mg/dL)
N	59.43 ± 17.06^a^	31.13 ± 15.55^a^	31.88 ± 12.77^a^	61.75 ± 6.20^a^	53.50 ± 7.29^a^
HFG	155.13 ± 52.78^b^	99.25 ± 48.48^b^	65.38 ± 23.24^b^	159.25 ± 15.88^b^	140.25 ± 26.25^b^
PTX	95.75 ± 18.07^c^	54.88 ± 14.33^c^	44.25 ± 6.88^c^	147.75 ± 35.26^b^	142.13 ± 40.06^b^

Abbreviations: N, normal group; HFG, high-fat diet plus hyperglycaemia group; PTX, high-fat diet/hyperglycaemia plus pentoxifylline treatment group; GOT, glutamate oxaloacetate transaminase; GPT, glutamate pyruvate transaminase; TG, triacylglycerol; TCHO, total cholesterol; HDL, high-density lipoprotein. Values are presented as means ± SD (N = 8); superscripted letters indicate significant differences (*P* < 0.05).
