# Supplementary material for: Pentoxifylline ameliorates non-alcoholic fatty liver disease in hyperglycaemic and dyslipidaemic mice by upregulating fatty acid β-oxidation
Source: Sci Rep. 2016 Sep 9;6:33102. doi: 10.1038/srep33102 (PMC5017161; doi:10.1038/srep33102)
Supplement: Supplementary Information [file srep33102-s1.pdf]

Pentoxifylline ameliorated nonalcoholic fatty liver disease in the hyperglycemia and dyslipidemia mice by the up-regulation of fatty acid β-oxidation.

Jia-Hung Ye[1], Jung Chao[2], Ming-Ling Chang[3], Wen-Huang Peng[4], Hao-Yuan Cheng[5], Jiunn-Wang Liao[6], Li-Heng Pao*[1,7,8]

[1]Research Center for Industry of Human Ecology, College of Human Ecology, Chang Gung University of Science and Technology, Taoyuan, Taiwan
[2]Institute of Pharmacology, College of Medicine, National Yang-Ming University, Taipei, Taiwan
[3]Liver Research Center, Division of Hepatology, Department of Gastroenterology and Hepatology, Chang Gung Memorial Hospital, Linko, Taiwan
[4]Department of Chinese Pharmaceutical Sciences and Chinese Medicine Resources, College of Pharmacy, China Medical University, Taichung, Taiwan
[5]Department of Nursing, Chung Jen College of Nursing, Health Sciences and Management, Chia-Yi, Taiwan
[6]Graduate Institute of Veterinary Pathology, National Chung Hsing University, Taichung, Taiwan
[7]Graduate Institute of Health-Industry Technology, College of Human Ecology, Chang Gung University of Science and Technology, Taoyuan, Taiwan
[8]Department of Nutrition and Health Sciences, Chang Gung University of Science and Technology, Kweishan, Taoyuan, Taiwan

*Address all correspondence to Li-Heng Pao
Email: paolhaa@gmail.com
Phone: +886-3-2118999 #5111
Fax: +886-3-2118866

Supplementary information

Western blot analysis

Total protein was extracted from the liver tissue using ice-cold T-PER extraction buffer (Thermo, USA) supplemented with protease inhibitor (Roche applied science, Germany). After the homogenization, debris was removed by centrifugation (12,000 rpm, 15 min, 4°C. The total protein concentration was quantified using a Protein Assay Reagent kit (Bio-Rad, USA) at 595 nm. Proteins (30 µg) were loaded into a gel prepared from TGX FastCast Acrylamide Solution (Bio-Rad, USA) and electrophoresed at 250V for 40 min.

Then, the total protein was transferred to a PVDF membrane (100V, 90 min, 4°C) and blocked with 5% non-fat milk in PBST for 1 hour at room temperature. The molecular weights of examined proteins were compared to those of pre-stained protein markers (Prep1025, Bioman, Taiwan), and the membrane was carefully cropped. Primary antibodies against PPAR-α (GTX101098, 1:1000, GeneTex), MCAD (GTX100488, 1:1000, GeneTex), PGC1α (GTX37356, 1:500, GeneTex), actin (MAB1501, 1:1000, Millipore), and GAPDH (MAB374, 1:1000, Millipore) were added to the cropped membrane. After overnight hybridization at 4°C, HRP-conjugated secondary antibodies (for rabbit: AP132P, 1:5000; for mouse: AP124P, 1:5000, Millipore) were added to the cropped membrane at room temperature for 1 hour. The cropped membrane was then developed using Clarity ECL Western Blotting Substrate kit (Bio-Rad, USA) and digitally semiquantified using a MultiDoc-It Imaging system (UVP, USA). Each band was quantified using VisionWorksLS software (UVP, USA), and the relative expression of the protein was calculated by dividing its band intensity by that of the loading control.

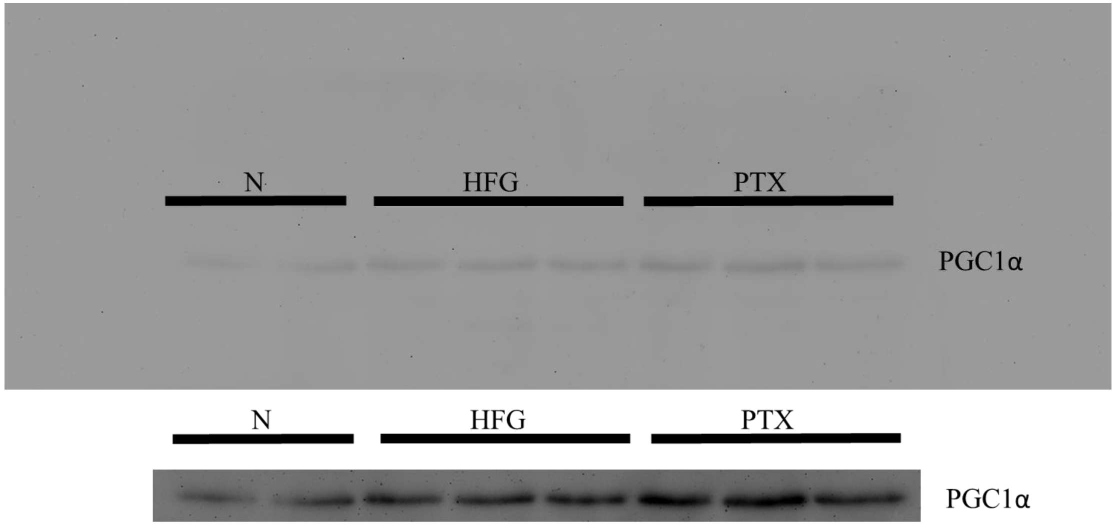

Figure S1. Different contrast of PGC1α protein.

The cropped and high contrast image of PGC1α was compared to its origin. Because the digital quantification using VisionWorksLS software was calculated from the original data, the quantification would not be affected by the adjustment of contrast.

NMR-based metabolomics data in blood, urine, and liver.

Supplementary Table S1: Metabolites in blood (N=8)

	N (mean ± sd)	HFG (mean ± sd)	PTX (mean ± sd)	p value (N vs. HFG)	p value (N vs. PTX)	p value (HFG vs. PTX)
**valine**	151.477063±20.148383	159.081838±42.730033	133.642455±22.727865	0.679232936	0.142916073	0.192654394
**lactate**	2818.858738±470.894749	6506.81145±706.915022	3198.36585±553.19802	6.72E-08	0.189130823	2.07E-07
**alanine**	205.107954±65.929914	268.676869±43.234805	172.424714±48.633685	0.05409308	0.310607712	0.001597979
**glutamine**	136.561273±12.131976	193.822995±37.512378	194.381134±22.898422	0.004446631	0.000117201	0.97376812
**citrate**	50.288941±17.983783	65.506903±17.568951	68.651278±8.100582	0.131601766	0.034116929	0.676444572
**choline**	228.42165±13.316997	399.6912±56.615949	270.518138±48.26305	6.18E-05	0.056533753	0.000444173
**glycine**	189.2394±20.449721	326.541013±51.436248	244.032975±29.313518	9.56E-05	0.001467504	0.003520522
**α-glucose**	444.473705±87.460532	1284.002591±287.661114	690.564306±171.257699	6.44E-05	0.006549265	0.000599196
**tyrosine**	10.759643±1.732602	19.774601±3.619765	13.41708±2.642688	0.000139794	0.04588782	0.002472081
**histidine**	5.790796±1.56357	6.908917±1.495971	8.049904±0.900389	0.193195709	0.006774978	0.110581395
**phenylalanine**	14.258727±3.849137	14.066461±2.054421	13.444495±3.992623	0.909347121	0.703539675	0.721307671
**formate**	2.542169±1.061847	4.438815±0.875767	2.970563±0.794841	0.002792456	0.408322196	0.005485067
**acetate**	65.019675±29.454656	65.6421±20.92653	49.093063±8.106655	0.964359837	0.204882035	0.08263594
**lysine**	66.036375±7.510144	88.933±5.694674	79.478613±3.467986	2.20E-05	0.001616252	0.002940138
**3-hydroxybutyrate**	276.666695±143.333516	1818.3854±1477.729417	1081.03065±319.485521	0.028091861	0.000134799	0.234543765

**leucine**	264.4007±35.044092	318.0622±74.472026	272.9457±38.039378	0.115381077	0.668741393	0.182735194
**creatine**	98.331929±18.511899	145.835799±14.659171	133.735928±8.101283	0.000128033	0.001038087	0.082565544
**pyruvate**	50.444288±5.877981	59.07675±12.161619	46.7263±2.238752	0.121438253	0.152315889	0.0314119
**acetoacetate**	59.908525±15.370267	130.751613±100.210871	122.9082±38.131555	0.105061085	0.00272929	0.850838541
**acetone**	104.925459±34.240889	452.998034±441.549817	238.978188±78.639616	0.075679646	0.002230756	0.244871605
**3-hydroxyisobutyrate**	27.659525±6.209137	42.587346±6.28537	23.939681±8.810097	0.000528638	0.378353683	0.00057335
**1,3-dihydroxyacetone**	33.948033±17.515598	169.542139±112.204134	88.264804±52.646152	0.014987579	0.030473985	0.113578112
**Acetoin**	57.787631±21.649574	106.119944±29.452596	61.714588±27.805725	0.003988582	0.772709194	0.011661734
**Cholesterol**	0.920389±0.172126	1.443277±0.145379	0.893459±0.139834	2.89E-05	0.752937965	4.52E-06
**Total FA**	145.712641±12.886296	260.692048±23.813756	246.178984±38.299983	2.76E-07	0.000127629	0.411621397
**PUFA**	27.837439±7.68773	73.171831±9.113633	93.511541±16.340068	1.12E-07	2.34E-06	0.015109396
**UFA**	29.106727±8.678571	88.635021±13.101492	76.369159±17.570645	3.14E-07	7.27E-05	0.162619615

**p <0.05 (N vs. HFG)**
lactate
glutamine
choline
glycine
α-glucose
tyrosine

formate
lysine
3-hydroxybutyrate
creatine
3-hydroxyisobutyrate
1,3-dihydroxyacetone
Acetoin
Cholesterol
Total FA
PUFA
UFA

**p <0.05 (HFG vs. PTX)**
lactate
alanine
choline
glycine
α-glucose
tyrosine
formate
lysine
pyruvate

3-hydroxyisobutyrate
Acetoin
Cholesterol
PUFA

Supplementary Table S2: Metabolites in urine (N=8)

	N (mean ± sd)	HFG (mean ± sd)	PTX (mean ± sd)	p value (N vs. HFD)	p value (N vs. PTX)	p value (HFD vs. PTX)
**Methylamine**	0.003396±0.000448	0.001162±0.000504	0.002211±0.000285	5.18E-07	7.47E-05	0.000554
**Methylmalonate**	0.00773±0.001065	0.006579±0.001764	0.007349±0.000505	0.166432	0.413198	0.298439
**NAG**	0.011807±0.000497	0.012964±0.004037	0.01108±0.000376	0.475852	0.008618	0.258152
**2-Oxoglutarate**	0.009364±0.003252	0.010395±0.009927	0.003934±0.000398	0.800221	0.002993	0.12887
**Succinate**	0.007252±0.000998	0.011841±0.020652	0.003883±0.000162	0.575427	3.58E-05	0.341892
**citrate**	0.033429±0.010671	0.01207±0.00667	0.007129±0.00156	0.000779	0.000293	0.093888
**creatine**	0.007598±0.00121	0.004305±0.001707	0.003522±0.001081	0.001183	1.18E-05	0.326032
**choline**	0.002797±0.000148	0.006831±0.003975	0.003324±0.000272	0.031286	0.000917	0.052364
**taurine**	0.01313±0.003233	0.015576±0.007227	0.011267±0.003136	0.433472	0.292338	0.179968
**Creatinine**	0.021879±0.001577	0.014519±0.0051	0.019069±0.002431	0.006082	0.024738	0.058847
**N-Phenylacetylglycine**	0.004776±0.001508	0.004266±0.001906	0.005828±0.000912	0.588288	0.141309	0.078979
**Allantoin**	0.018918±0.002777	0.013651±0.004535	0.014318±0.002829	0.022922	0.008322	0.747209
**Sarcosine**	0.012061±0.00092	0.018194±0.006538	0.006906±0.001036	0.042348	1.28E-07	0.00244
**1-methylnicotinamide**	0.000647±0.000092	0.000438±0.000218	0.000371±0.000127	0.043047	0.00049	0.499969
**Hippurate**	0.011179±0.001552	0.001017±0.000471	0.001162±0.000137	1.22E-07	5.08E-07	0.458191

**Trigonelline**	0.002548±0.00032	0.000632±0.000504	0.00109±0.000235	2.21E-06	2.79E-07	0.054527
**Succinylacetone**	0.004781±0.000606	0.008889±0.006555	0.007899±0.000876	0.142067	4.16E-06	0.703649
**Tartrate**	0.002598±0.00008	0.019157±0.006054	0.020844±0.00467	0.000172	1.71E-05	0.569218
**Nicotinamide N-oxide**	0.000443±0.000062	0.000242±0.000127	0.000324±0.000063	0.003647	0.003042	0.159177
**3-Indoxylsulfate**	0.001024±0.000265	0.0003±0.00017	0.000593±0.000163	5.71E-05	0.003462	0.005304
**TMA**	0.053806±0.005666	0.005223±0.003402	0.009012±0.005328	3.88E-10	4.36E-10	0.13902
**2-Oxoisocaproate**	0.017876±0.000858	0.015843±0.004042	0.018813±0.000841	0.231076	0.058083	0.095455
**3-Hydroxyisovalerate**	0.002636±0.000336	0.00185±0.000454	0.002435±0.000148	0.002802	0.179265	0.01099

**p <0.05 (N vs. HFG)**
Methylamine
citrate
creatine
choline
Creatinine
Allantoin
Sacrosine
1-methylnicotinamide
Hippurate
Trigonelline
Tartrate
Nicotinamide N-oxide

3-Indoxylsulfate
TMA
3-Hydroxyisovalerate

**p <0.05 (HFG vs. PTX)**
Methylamine
Sacrosine
3-Indoxylsulfate
3-Hydroxyisovalerate

Supplementary Table S3: Metabolites in liver (N=8)

	**N (mean ± sd)**	**HFG (mean ± sd)**	**PTX (mean ± sd)**	**p value (N vs. HFG)**	**p value (N vs. PTX)**	**p value (HFG vs. PTX)**
**Bile acid**	9.237383±11.10709	9.959294±4.751008	5.93168±1.83579	0.877691	0.461388	0.065802
**Valine**	93.424003±37.625597	48.509076±10.026251	40.667217±15.757472	0.015803	0.007157	0.288618
**Isoleucine**	30.682529±13.155135	13.502879±2.868975	12.557069±5.445121	0.010333	0.007868	0.692398
**Leucine**	32.239338±14.540334	13.587217±2.747444	12.665442±5.21194	0.011313	0.008803	0.687166
**3-Aminoisobutyrate**	3.124627±0.601189	5.556255±6.948434	2.593136±0.319643	0.386551	0.064079	0.296709
**3-hydroxybutyrate**	16.15415±8.483008	79.123071±64.30361	37.810053±14.090853	0.036029	0.004807	0.137031
**Lactate**	313.389478±128.521151	494.346368±95.3297	391.326248±144.215533	0.010465	0.304066	0.140569
**Alanine**	164.078935±57.504561	123.631882±16.895426	97.947653±36.662999	0.111098	0.024907	0.123699

**Glutamate**	46.765611±22.196418	34.551855±8.053769	27.26937±12.29677	0.205016	0.067099	0.214322
**Succinate**	23.405641±10.773193	57.155428±14.782433	33.872765±13.501664	0.000313	0.132274	0.008266
**Glutamine**	18.104751±7.67775	57.86664±9.147529	39.977954±14.389534	5.49E-07	0.004771	0.01694
**Aspartate**	6.403869±3.749342	2.974566±1.206667	2.970984±1.252305	0.048579	0.048663	0.995729
**GSH**	42.282234±14.259133	40.348152±9.416817	30.835545±10.947885	0.769661	0.115675	0.103757
**Choline**	55.620043±23.25119	33.89974±12.834972	38.294994±14.642649	0.053565	0.12158	0.560061
**Carnitine**	50.792841±17.020283	40.963564±11.011686	38.40247±16.137061	0.223794	0.184018	0.734543
**Taurine**	231.185219±78.185206	250.844763±40.980947	227.030199±78.790842	0.568077	0.922513	0.493454
**Glycine**	95.464425±31.816092	123.460275±26.633089	86.881486±31.385325	0.096579	0.619285	0.034324
**α-glucose**	106.912901±48.987633	295.324413±197.816236	222.259779±84.404976	0.040716	0.0094	0.391034
**β-glucose**	64.151746±29.582596	195.427152±101.512318	134.011865±52.145911	0.01077	0.010331	0.183656
**Fumarate**	4.717496±1.427865	3.825122±1.086586	3.008007±0.995547	0.210838	0.022652	0.164649
**Tyrosine**	8.303055±2.825916	4.091458±0.601557	3.495844±1.486767	0.005284	0.002308	0.350882
**Histidine**	5.898088±1.802047	4.05522±1.099491	3.247144±1.183057	0.040227	0.006849	0.206907
**Uridine**	7.008911±2.569668	7.40123±1.29832	4.715397±1.53097	0.725697	0.066482	0.003388
**Xanthine**	7.862706±2.571603	3.956767±1.990064	4.911119±1.682248	0.007164	0.02579	0.349448
**Hypoxanthine**	4.744958±2.384408	2.681717±1.010877	2.30709±0.767326	0.062894	0.031503	0.448738
**Inosine**	0.87722±0.298169	0.575057±0.40951	0.255129±0.255405	0.138919	0.000949	0.105521
**Adenosine**	21.652193±7.04291	16.972572±6.960548	11.645704±4.439472	0.23167	0.008071	0.113763
**Formate**	1.453653±0.484197	1.060612±0.78187	1.188439±0.835733	0.280849	0.482417	0.77196
**AMP**	0.946712±0.493028	1.622406±0.735315	1.085907±0.559417	0.065912	0.629165	0.148309
**Nicotinurate**	5.901801±1.853034	4.703946±1.190852	3.664124±1.242719	0.175904	0.020748	0.132317

**Cholesterol**	8.436046±0.708976	12.377257±3.530045	9.91826±1.936297	0.021246	0.090204	0.134766

**p <0.05 (N vs. HFG)**
Valine
Isoleucine
Leucine
3-hydroxybutyrate
Lactate
Succinate
Glutamine
Aspartate
α-glucose
β-glucose
Tyrosine
Histidine
Xanthine
Cholesterol

**p <0.05 (HFG vs. PTX)**
Succinate
Glutamine
Glycine

Uridine
